# Trophic factors intervention regenerates the nestin-expressing cell population in a model of perinatal excitotoxicity: Implications for perinatal brain injury and prematurity

**DOI:** 10.15761/imm.1000228

**Published:** 2016-06-25

**Authors:** A. Espinosa-Jeffrey, R. A. Arrazola, B. Chu, A. Taniguchi, S. M. Barajas, P. Bokhoor, J. Garcia, A. Feria-Velasco, J. de Vellis

**Affiliations:** Semel Institute for Neuroscience and Human Behavior, David Geffen School of Medicine at UCLA. Intellectual and Developmental Disabilities Research Center, Neuroscience Research Building, 635 Charles E. Young Drive South, Los Angeles, California 90095-7332, USA

**Keywords:** excitotoxicity, oligodendrocyte, PVL, myelination, transferrin/IGF-1

## Abstract

We previously showed that TSC1 (a combination of transferrin and IGF-1) is a potent inductor of myelinogenesis in myelin deficient rats and in demyelinated adult mice. More recently, we demonstrated that regeneration of oligodendrocyte progenitors and myelin are possible with a single dose of TSC1 in a mouse model of Premature birth. Here, using the same mouse model of perinatal white matter damage due to glutamate excitotoxicity (GME), we tested the hypothesis that regeneration of endogenous nestin-expressing neural progenitors improves the outcome of prematurity. Treatments: N-methyl-D-aspartate (NMDA), saline, NMDA+TSC1 together or NMDA followed byTSC1 3 days later, were stereotaxically delivered into the corpus callosum of P4 mouse pups. Fluorescence analysis showed an intense enrichment of nestin-expressing cells in groups injected with NMDA+TSC1 from which many were generated by proliferation. Moreover, when TSC1 was injected three days after the primary insult it was still able to reduce ventricular enlargement and extensively rescue nestin-expressing progenitors. Cells co-expressing the proliferation marker Ki67, CNPase and faint nestin label were more abundant in groups injected with MNDA+TSC1 at 35 days after injection. Stereological analysis showed that the number of nestin-expressing cells in the sub-ventricular zone correlated inversely with the volume of the ventricle. A delayed administration of TSC1 after excitotoxicity reduced ventriculomegaly but not as much as, when NMDA and TSC1 were injected simultaneously. Thus, the earliest TSC1 was administered, the more tissue was rescued as shown by reduced ventriculomegaly. Astrocytes responded to GME by upregulating the expression of estrogen receptor and this expression was attenuated in the presence of TSC1 suggesting a decreased inflammation and a lesser need for estrogen-mediated central nervous system (CNS) neuroprotection.

## Introduction

Perinatal brain injury is a common cause of life-long neurological disability. The etiology is complex and multifactorial including hypoxia-ischemia, inflammation, and excitotoxicity being all of them key factors of perinatal brain injury (BI). Neonatal care has dramatically improved the survival rate after premature birth yet, there still remains significant neurological morbidity associated with prematurity. In the United States, about 500,000 or approximately 12.5% of neonates are premature babies [1]. Approximately 5–10% of infants born <32 weeks gestational age weight <1500g or less (very low birth weight, VLBW) showing major motor deficits and up to 60% have neuro-cognitive deficits and/or behavioral and intellectual life-long disabilities [2–3]. A unique pattern of injury observed in more than 50% of VLBW infants is periventricular leukomalacia (PVL), presents diffuse cerebral white matter damage with injury to premyelinating oligodendrocytes (OL). White matter injury is associated with neuronal and axonal abnormalities and it is known as encephalopathy of prematurity (EOP) [4]. Diagnosing PVL is important because a significant percentage of surviving premature infants develop cerebral palsy (CP), intellectual disabilities and/or visual disturbances.

Whether peri-ventricular white matter injury occurs due to prematurity, peri-natal maternal infection or inflammation the resulting early injuries disrupt normal brain development and alter its maturation leading to developmental and behavioral deficits to which currently there is no cure.

Upon insult eukaryotic cells have complex mechanisms for achieving survival. Among these are heat shock proteins or stress proteins (HSPs). These proteins are induced not only by heat but also by a variety of other physiological stressors including inflammatory cytokines and mediators [5–6]. HSPs are found in the cytoplasm, mitochondria, peroxisomes and endoplasmic reticulum and they ensure appropriate protein folding. Stress proteins serve as indicators that cells have been or are in a stress situation underlying the pathogenesis of developmental and/or degenerative diseases and disorders of the CNS. Stress proteins are classified based on their molecular weight giving rise to 5 major classes of HSPs (100, 90, 70, 60 and the small HSPs with molecular weights ranging from 12 to 43KDa that includes the α-crystallin and HSP-32 family of proteins. HSP-32 was first observed in cells exposed to heavy metals and it was characterized as the microsomal enzyme hemoxygenase-1 (HO-1) [7–10]. This hemoxygenase isoenzyme is also induced by reactive oxygen metabolites (ROM) [6–10]. HSP-32 expression is low in the normal brain [11]. After ischemic insult or heat shock, neurons and glia have increased levels of this protein [11, 12]. OL have a low basal expression of HSP-32 [13] that is upregulated in response to oxidative stress and severe hypothermia [14] but not by heat shock [15].

Estrogen plays a critical role in the development, maintenance, and physiology of male and female reproductive tissues as well as non-reproductive systems, including the cardiovascular, skeletal, and central nervous system (CNS) [16]. Estrogen mediates its effects by binding to estrogen receptor α (ERα) and ERβ, members of the steroid receptor nuclear receptor super family. ERs bind to their ligand dimerization to further interact with DNA sequences that modulate gene transcription [17,18]. More recently, new evidence indicates rapid, nongenomic actions of estrogen that appear to be mediated through extranuclear ERα and/or ERβ [19–22]. The CNS is a major target of estrogen activity [23,24], yet the mechanisms by which estrogen mediates its effects are not clear. [25–28]. It has been shown that across species there are striking differences on the patterns of ERs gene expression [29], mouse [30], and human [31,32] and therefore, data cannot be extrapolated from one species to another. Estrogen’s neuroprotective effects have also been shown in experimental autoimmune encephalomyelitis (EAE) and other animal models. These effects are mediated through estrogen receptor a (ERa) in astrocytes [33]. These authors showed by conditional deletion of the receptor that signaling through ERa is essential for the beneficial effects of estrogen in EAE.

To date, no treatment leading to a cure has been developed for PVL. However, rodent studies implicate glutamate receptors as potential therapeutic targets [34]. In particular, excitotoxicity via the over-activation of ionotropic glutamate receptors including the α-amino-3-hydroxy-5-methyl-4-isoxazole-propionic acid glutamate receptors (AMPARs), N-methyl-D-aspartate receptors (NMDARs), and kainate receptors [35]. Reports the mechanisms leading to the pathophysiology are opening doors to new interventions. We previously showed that TSC1 (a combination of transferrin and IGF1) is a potent inductor of myelinogenesis in myelin deficient rats [36] and in demyelinated adult mice where a single treatment with TSC1 was sufficient to reverse hind-limb paralysis [37]. More recently we have shown the effects of TSC1 on the regeneration of the OL progenitor population and concomitant reduction of white matter loss [38]. Here addressing only one component of premature birth, excitotoxicity induced through the NMDAR we examined the neuroprotective effects of TSC1 on nestin-expressing progenitors. We used HSP-32 as a stress marker, and the pattern of expression of the estrogen receptor together with cell specific markers to understand how nestin-expressing progenitors and astrocytes are responding to this insult.

## Perinatal white matter injury (WMI)

It is the main target of hypoxia-ischemia in the perinatal brain. WMI is a leading cause of functional and cognitive disability, and is detected in a significant percentage of preterm infants. Injured immature brains exhibit a wide spectrum of lesions known as periventricular leukomalacia (PVL). In preterm neonates, PVL is the most common type of brain lesion following hypoxia-ischemia leading to cerebral palsy, cognitive deficits and life-long disabilities. Not only oligodendrocyte (OL) progenitors are the main target of this type of insult. Here we demonstrate that nestin-expressing neural stem cells that would be the source of new OLs is also impacted in the premature neonate. We also show that ventriculomegaly is substantially mitigated with TSC1 in a single treatment. This regeneration occurs both via neuroprotection of pre-existing OL progenitors and nestin-expressing cells at the time of injury and, also via proliferation and migration of nestin-expressing cells. This work together with our previous report on the effects of TSC1 on myelination [38] appear to be the first approach that addresses white matter repair by nestin-expressing cells using two natural molecules existing in the brain whose stoichiometry appears to be critical in postnatal brain development.

## Material and methods

### Animals and intraparenchymal injection procedures

Nestin-GFP mice were bred at the University of California Los Angeles (UCLA) in a restricted access, temperature-controlled vivarium under a 12 hr:12 hr light: dark cycle, with access to food and water *ad libitum*. Postnatal day four (P4) nestin-GFP mice pups were used. Each experiment consisted of three time points and five conditions: Non-treated mice, Saline, NMDA, NMDA+TSC1 simultaneously injected (N+TSC1sim) and NMDA + injection of TSC1 delayed 3d (N+TSC1 3d). The stereotaxic intraparenchymal injections in brain, collection and sectioning of tissues for the characterization of cell phenotypes were performed as described [36]. Assessment of cellular stress was monitored through HSP-32 expression. We used 6 mice per condition. Three separate experiments were performed to assess the effects of NMDA alone or with TSC1 on endogenous OLPS survival, proliferation and maturation. Intraparenchymal injections were performed according to previously described methods [36–38].

Briefly, nestin-GFP P4 pups were injected as follows. Ophthalmology microsurgery instruments and a stereotaxic apparatus with an adjustable adapter for small (newborn) animals were used. The animals were anesthetized with halothane and placed on a stereotaxic frame for the intraparenchymal administration of the cells with a Hamilton syringe. The injection coordinates were 1.2 mm lateral (right) and 0.7 mm caudal to Bregma. The needle was inserted 2.5 mm deep into the corpus callosum. The control group consisted of nestin-expressing mice that received saline injections, using the same procedures. We performed unilateral grafts. The total volume of the injected factors was 1.5 μl.

### Induction of GME

We used an animal model of PVL where the perinatal brain injury was induced by injecting NMDA into P4 nestin-GFP mice pups. Stereotaxic injection of NMDA was performed on P4 mice as described for administration of factors (see below).

### Preparation of trophic factor cocktail

Stock solutions were prepared in saline: TSC-1 was also prepared in salinewhere the final concentration was 100ng of IGF1 and 15 μg of apo-transferrin (Tf without iron) in 2 μl of saline.

### Animals and intraparenchymal injections

P4 mice pups were used for these experiments. Each experiment consisted of five conditions: Non-treated mice, Saline, NMDA, NMDA+TSC-1 simultaneously injected (N+TSC-1 0d) and NMDA + injection of TS1 delayed 3d (N+TSC-1 3d). n = 4 to 6 mice were used per condition. The Nestin-GFP transgenic mice were housed at UCLA in a restricted access, temperature-controlled vivarium on a 12hr:12hr cycle, food and water ad libitum. The stereotaxic intraparenchymal injections were performed as described [36]. Treatments were delivered into the corpus callosum (CC). The tip of the needle was labeled with charcoal prior to the injection to locate the injection site afterwards. Brain collection and sectioning for characterization of cell phenotypes was performed as described [36]. Animals were kept at least two months after treatment and behavioral assessments were performed 3 times a week. Assessment of cellular stress was monitored by HSP-90. Acute experiments were performed with 350 microns brain slices.

### Collection and examination of the samples

Brain collection and sectioning for the characterization of cell phenotypes was performed as previously described [36]. To determine the effectiveness of the treatment, in combination with cell and stage specific markers for OLs.

### Immunofluorescence and image analysis

Frozen sections were rinsed three times in PBS prior to immunocytochemistry. The primary antibodies included monoclonal anti-Ki67 IgG antibody (Vector VP-K451); anti-CNPase monoclonal IgG antibody (Chemicon MAB32R); supernatant of Melita Shacner’s O4 monoclonal antibody against sulfatides (a gift from Pfeiffer’s lab); antiHSP-32 monoclonal IgG antibody (Stress Gene OSA-110) and anti-caspase-9 antibody (Novus bio, NB100–56118); Anti-estrogen-α receptor antibody (ERα Antibody - C-311: sc-787 Santa Cruz, Biotechnologies). Normal mouse IgG and IgM as well as normal rabbit antibodies were used as control serum. The immunofluorescence procedures were performed as previously described [36–38]. Briefly, brain sections were rinsed in PBS. Samples were blocked for 1 h in 1% BSA (Sigma-Aldrich, Buchs, Switzerland), 0.3% Triton X-100 (Sigma-Aldrich), and 10% normal goat or donkey serum in PBS. Primary antibodies were diluted in carrier solution (1% BSA and 0.3% Triton X-100 in PBS) and incubated overnight at 4°C. After washing with PBS, cells were incubated with the appropriate secondary antibodies (1:200; Jackson Immuno-Research), for 1 h at room temperature washed with TBS and mounted onto slides with Vinol prepared in our laboratories). Samples were imaged using the LSM 510 META confocal microscope (Zeiss) and analyzed with the Axiovision software (Zeiss).

### Stereology: counting nestin-expressing cells

We used the Axio-Imager M2 (Zeiss) microscope. Quantification of nestin-expressing cells in mice was performed using the Optical fractionator Method (Stereo-Investigator, Micro-bright-field, mbf). Two regions of interest were selected, the SVZ and the CC. The entire sub ventricular region (3rd ventricle) and the corpus callosum were examined separately with the 63X oil-immersion objective. Fifty-one fields per region were selected randomly by the program. The cells were counted directly in a known fraction of each zone using the optical dissector in a systematic random pattern and the motorized stage from Zeiss. The cells were counted when the nucleus was clearly seen in focus and inside the counting frame. The area of each frame was 352 – 384 μm^2^. Only GFP bearing cells were counted.

### Ventricle area across treatments

The area of the ipsilateral ventricle was obtained with the Cavalieri Probe from micro bright field (mbf) the n15 to 22 fields per section were visited for these measurements.

### *Ex-vivo* experiments

Coronal brain slices (300μm) were used for the acute treatment of TSC1. After cutting a single section was placed in a 30mm Petri dish and observed with the LSM 510 confocal microscope using the 488 laser beam. Images were acquired every 2.5 min.

### Statistical analysis

Quantitative data is expressed as the means ± SE. Comparison of mean values between multiple groups was evaluated using ANOVA One-Way Analysis of Variance followed by a post hoc Tukey HSD multiple comparison test where data obtained with the various treatments were compared either to their respective saline control or non-treated samples within the same age group. Significance was assumed when P<0.05. Different color of asterisks indicates the correspondent group and represents the significance with respect to control within the same group. The data from the behavioral study were evaluated using ANOVA followed by Kruskall Wallis multiple comparison test.

## Results

### Assessing the extent of damage and potential for recovery: expression of HSP-32

**Figure 1.**
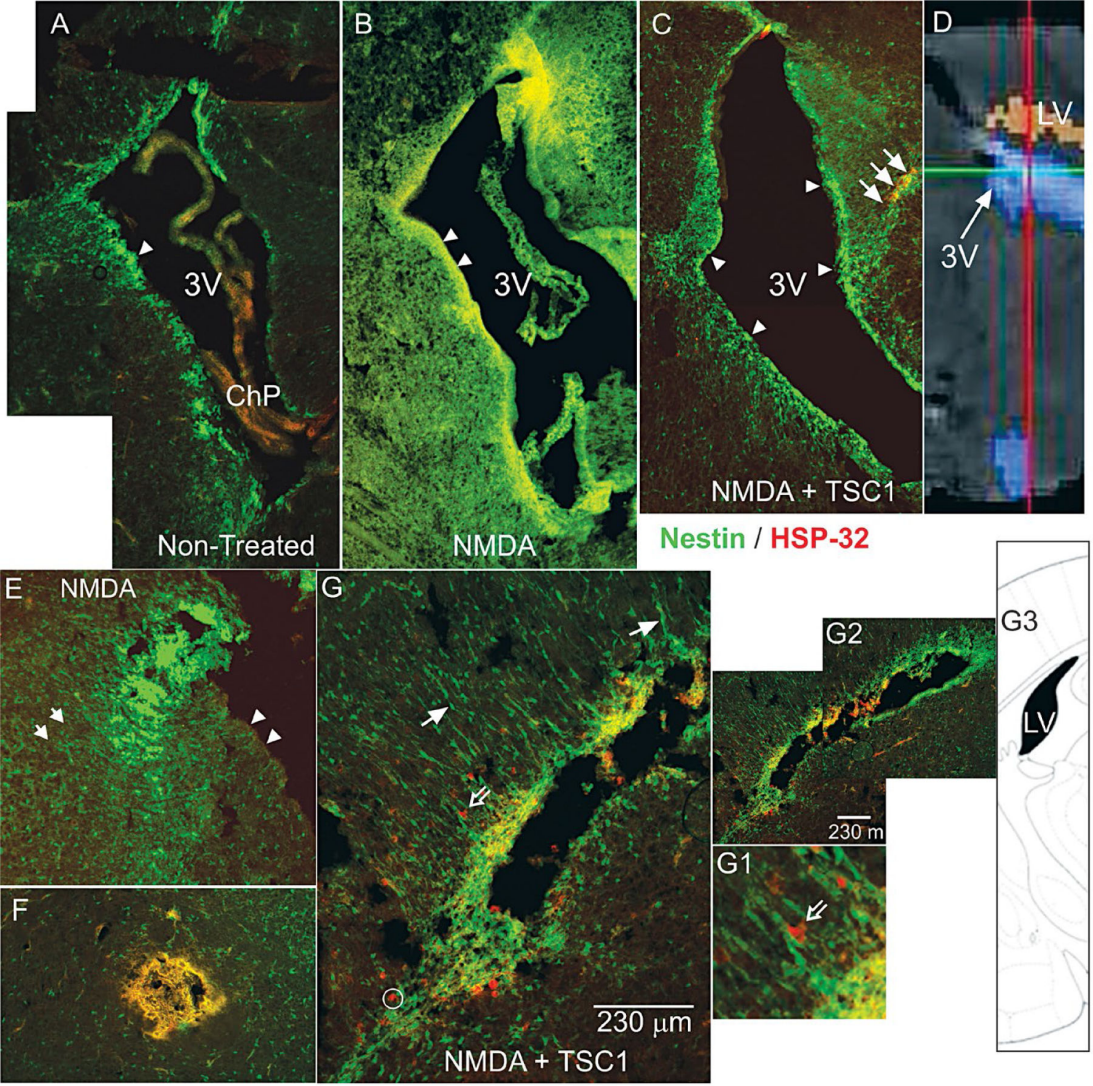
Low magnification views of para-sagittal (A-C/24h PI) and coronal (D,D1, D2 24h PI) brain sections from nestin-GFP mice immunostained for HSP-32. A) Non-treated animals at 6 days of age. The wall of the third ventricle and subventricular (SVZ) regions display migratory nestin-expressing cells (arrowheads) and an absence for HSP-32. B) The brain of an animal treated with NMDA at P5 and analyzed at P6 shows large cyst consisting of nestin positive and HSP-32 negative cells. Moreover, the wall of the third ventricle (arrowheads) as well as the SVZ appear to have lost the nestin expression. C) The brain of an animal treated with NMDA+TSC1 at P5 and analyzed at P6 displays an intact ventricular wall (arrowheads), and a thicker SVZ in all areas. More nestin positive cells appear to migrate towards the parenchyma with a few co-expressing HSP-32 (arrows) versus non-treated animals. D) Diagramatic view of the 3^rd^ ventricle of the mouse brain. E) View of another animal 24 hours after NMDA injection, where the wall of the ventricle was deprived of nestin positive cells (arrowheads). Clustered nestin-expressing cells accumulated near the ventricle (arrow). Only single scattered nestin-expressing cells were seen in the vicinity. F) In the brain parenchyma a cyst had formed from cells that appeared to co-express nestin and HSP-32 giving an orange color. The parenchyma had nestin-expressing cells that appeared to be organized in a concentric pattern with respect to the cyst. These clustered cells might tend to disappear with time. G) Coronal view of a mouse treated with NMDA+TSC1 where there were numerous “radial glia-like” nestin positive cells with long processes oriented perpendicular to the wall of the ventricle (solid arrows) and along these fibers nestin-negative/HSP-32 positive cells appeared to migrate (open arrows, (G1insert). G2). Low magnification view of the same ventricle showing extensive nestin expression. G3) Diagramatic representation of a coronal view of the mouse brain showing the lateral ventricle (Figure 1E).

The excitotoxic effects of NMDA were visible already 24 h after injection, the ventricle was already enlarged (Figure 1B) with respect to brains from mice injected with saline or NMDA+TSC1. The saline injection did not have any adverse effect, the size of the ventricle appeared normal. Nestin-GFP expressing progenitors were nicely distributed around the ventricle and in the subventricular zone (SVZ). Nestin positive cells appeared to be migrating from the subventricular zone (SVZ) into the brain parenchyma (Figure 1A). In contrast brains that received the NMDA injection, aside from ventricular enlargement, had acquired a spongy texture, and nestin-expressing cells were basically absent from the wall of the ventricle, the SVZ and surrounding parenchyma (Figure 1B). When NMDA was co-injected with TSC1, the appearance of the brain was much healthier and the ventricle was slightly enlarged. Nonetheless, nestin-expressing cells were abundant in the wall of the ventricle and the SVZ. These cells appeared to be migrating into the parenchyma in a similar manner to brains injected with saline (Figure 1C). The same brain sections were immunolabeled to detect the stress protein HSP-32; saline injected mice did not show any HSP-32, neither those receiving NMDA injections. In contrast, the NMDA+TSC1 treatment elicited a robust expression and migration of nestin positive cells and HSP-32 expression in some nestin positive cells; many of them located at the level of the ventricular wall and in the SVZ as well as in a few migrating cells in the SVZ. Nonetheless, most nestin positive cells did not express HSP-32 in spite of their location. Some nestin negative/HSP-32 positive cells were also found mostly in the parenchyma adjacent to the SVZ. In NMDA+TSC1 injected animals the ventricular wall and SVZ were intact and populated by nestin-expressing cells. They were bipolar cells migrating perpendicularly to the ventricular wall into the brain parenchyma. We also found nestin negative cells that were HSP-32 positive after injection (Figure 1C and 1D). Some of these cells appeared to migrate on nestin labeled radial glia-like cell fibers as shown (Figure 1D and D1 open arrow). By 7 days after injection no HSP-32 expression was found. HSP-32 was expressed by few of the cells within the clusters (data not shown). These cysts of progenitors were seen in the parenchyma or adjacent to the ventricle and were not present at later time points.

### Ventriculomegaly was reduced by a single trophic factor intervention

**Figure 2.**
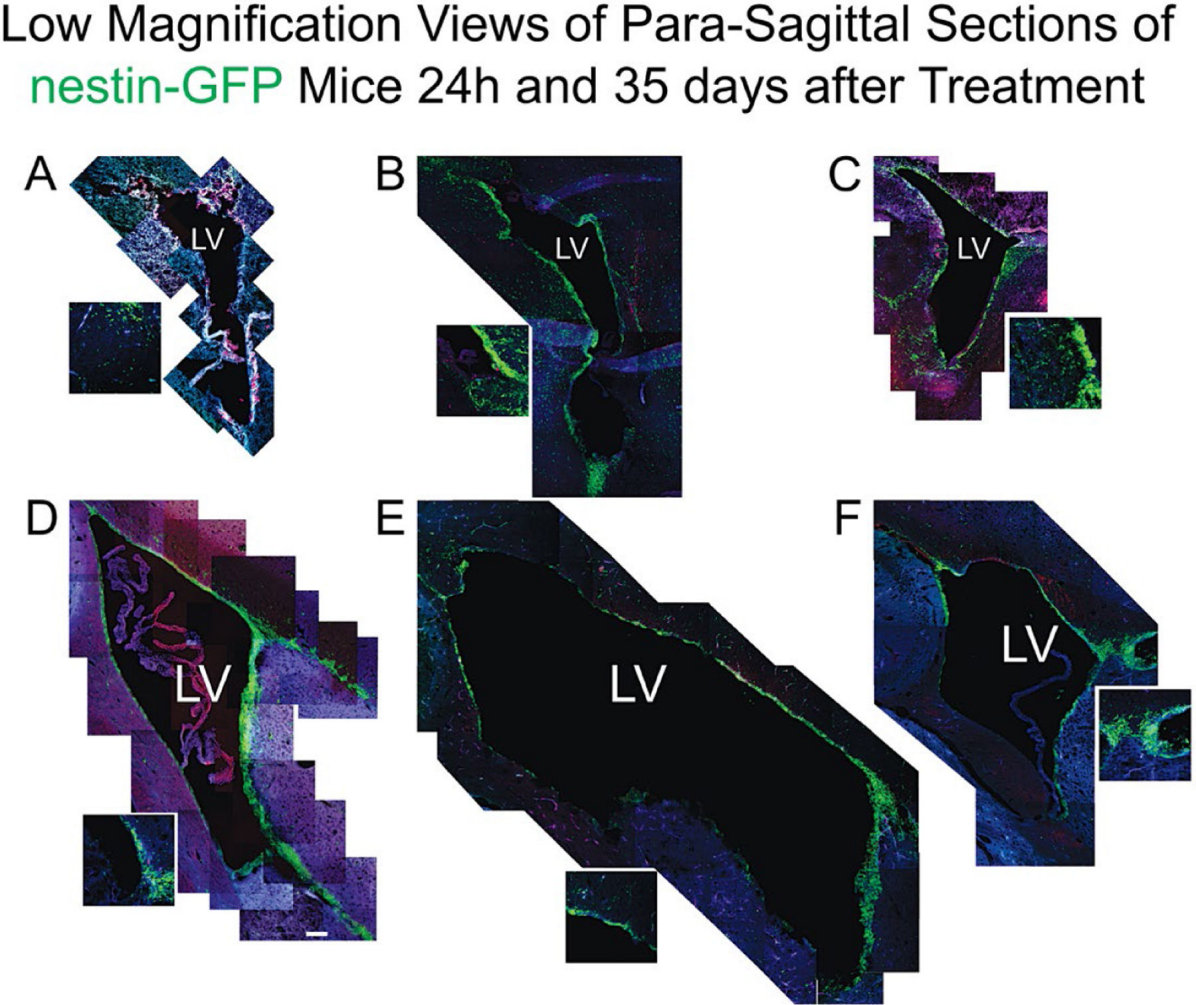
Low magnification views of para-sagittal sections of nestin-GFP transgenic mice24 hours after Treatment. Sections were immunolabeled forKi67 and CNPase. A) Non-treated animal 6 days of age or D) non-treated mouse 35 days of age. B) Twenty-four hours after NMDA injection the ventricle of mice injected with NMDA was already visibly enlarged with respect to non-treated animals and those that received the NMDA+TSC1 combination. The wall of the ventricle and subventricular regions displayed nestin+, Ki67+ and CNP+ cells. In many cases nestin was co-expressed with Ki67 (yellow cells) indicating that they were originated *via* cell proliferation (insert). D) View of a non-treated mouse thirty nine days of age where the inner portion of the ventricle had just a few nestin+ cells scattered along the wall of the ventricle and those from the SVZ had migrated away from this region. The outer portion of the ventricle was still thick but almost no nestin positive cells were seen beyond the SVZ. E) At this time point NMDA injected mice had a very large ventricle where the inner portion of the wall had very few nestin positive faintly labeled cells. Almost no cell expressed Ki67 nor CNPase in this region. F) The ventricle of mice treated with NMDA+TSC1 was much smaller than in mice without TSC1 treatment. The outer portion of the ventricle still displayed nestin+ cells and some appeared to migrate from the SVZ to the parenchyma. Many of these cells (co-expressed Ki 67 (insert). Cells expressing ki67 alone were not present in the vicinity of the ventricle at this time point suggesting that they may have had migrated.

The size of the third ventricle was differentially affected across treatments. Figure 2 illustrates the comparative images of the full ventricle taken from brains from mice 6 days of age (non-treated mouse) or either NMDA alone or NMDA+TSC1 injections. The comparative figure composite shows that as early as 24h after injection of NMDA alone the ventricular enlargement was evident for mice receiving NMDA alone and the enlargement was reduced when NMDA was injected in the presence of TSC1. Other differences at this time point, included the distribution of nestin-expresing cells between NMDA injected alone or combined with TSC1, where the outer portion of the ventricle was devoid of nestin+ cells in NMDA injected mice, while mice treated with TSC1 displayed nestin+ cells around the ventricle and their migration into the SVZ and the parenchyma appeared to be undisturbed by NMDA. Many nestin-expressing cells emerging from the ventricular wall and extending into the SVZ displayed ki67, a marker for proliferation; this is more visible in the inserts accompanying the low magnification images. Nestin negative Ki67 positive cells were also observed on the surrounding parenchyma. Thirty-five days after the intervention, the difference between the brains from mice receiving NMDA alone and those receiving NMDA+TSC1, was basically the size as shown (Figure 2).

### Measurement of the ipsi-lateral ventricular area

**Figure 3.**
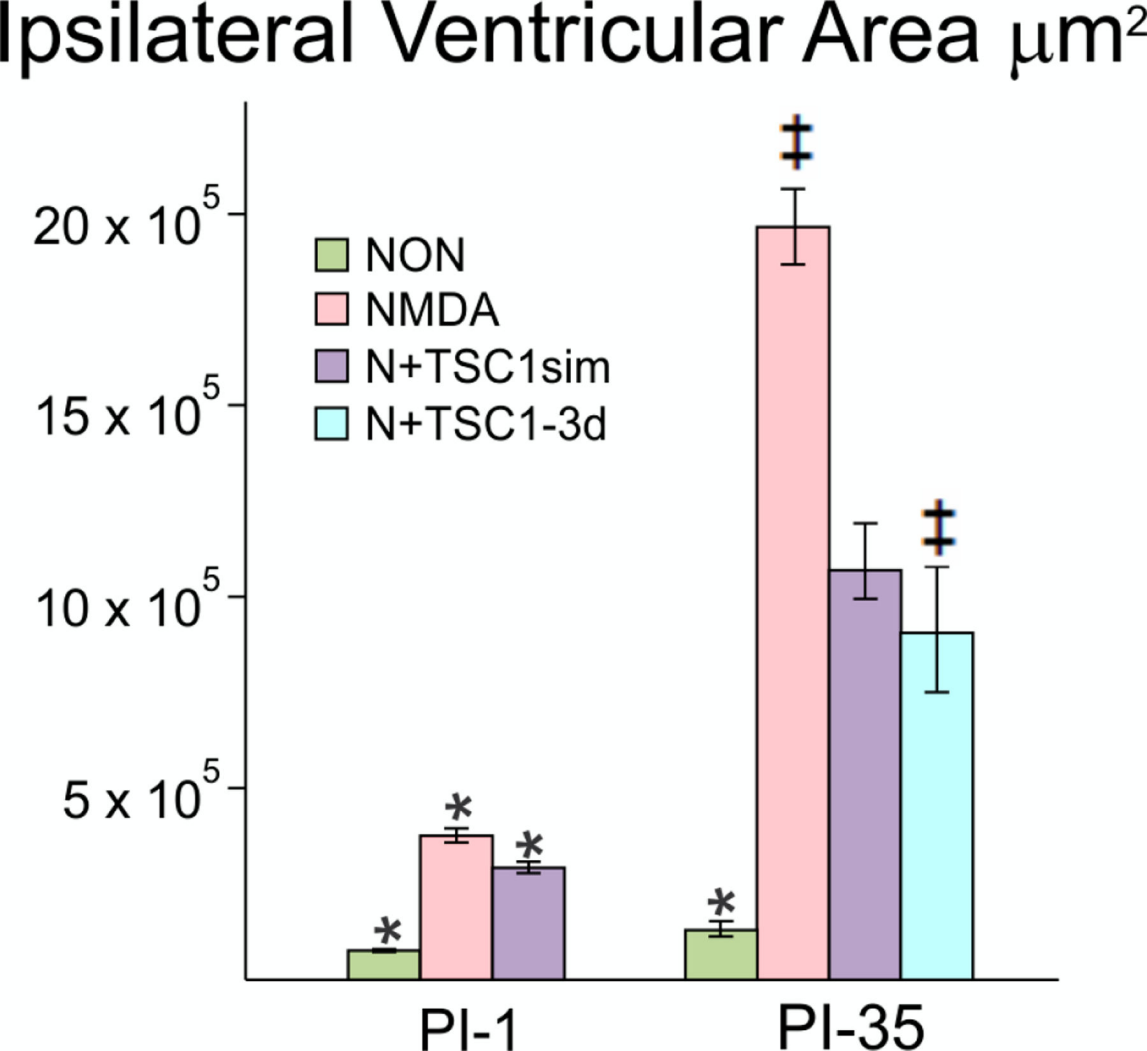
The enlargement of the Ipsilateral ventricle was differentially impacted across treatments. Using stereology we next measured the ipsilateral portion of the 3^rd^ ventricle starting from the midline. This morphometric analysis differs from the above measurements in that here only the ipsilateral portion of the ventricle was measured and compared across treatments at a given time point as opposed to the comparison of the area of the lateral ventricle in the injected hemisphere versus the non-injected (contralateral) one in the same brain section: NMDA alone (pink bars); NMDA+TSC1 injected simultaneously (purple bars) and NMDA followed by delayed treatment of TSC1 (turquoise bar). The third ventricle suffered a dramatic tissue loss as a function of time in mice injected with NMDA alone. Thirty-five days after the simultaneous injection of NMDA + TSC1 the enlargement of the third ventricle was visible but it was around 8 times larger than in the non-treated mice. The same was true for mice receiving the delayed injection where the ventricular size was around 50% smaller than in mice injected with NMDA alone. In the PI-1 group, the differences across treatments were statistical significant. In the PI-35 group mice treated with TSC1 simultaneously and those that received TSC1 3 days after NMDA the differences were not significant suggesting that treatment with TSC1 even three days after excitotoxicity brain tissue will be spared; while with respect to NMDA alone cell numbers were significant. Values are expressed as mean ± SEM; *p<0.05, ^‡^p<0.01 comparison versus their respective control.

Using stereology, we next examined para-coronal sections to measure the ventricular size of GFP-nestin transgenic mice either non-treated or injected with NMDA alone, NMDA + TSC1 injected simultaneously N + TSC1 sim, or NMDA followed by a delayed (3 days) injection of TSC1 (N+TSC1–3d). Frozen sections (28 μm) were cut, washed, and mounted to be examined under the AxioVision microscope (Zeiss) equipped with the Stereo Investigator software (MBF). The area of the ventricle was obtained using the Cavalieri Probe (MB field) (Figure 3). The values from six adjacent coronal sections per time point, per mouse, were obtained from three separate experiments. The mean values of these six sections were considered the value per mouse, per treatment for its respective experiment. Seven days after intraparenchymal injections, the animals treated with NMDA alone showed larger ipsilateral ventricle. When NMDA was injected with TSC1 simultaneously (N + TSC1sim), a significant reduction of around 50% in the ventricle area was observed (Figure 3). We also examined whether a delayed injection of TSC1 would increase the neuroprotective effect if injected after cell death and the active inflammatory events that would occur within hours after the insult. We observed that the area of the ventricle was reduced even more (under 50% of the NMDA treated mice) but this difference was statistically non-significant (Figure 3).

### Neuroprotection of nestin-expressing cells by TSC1

**Figure 4.**
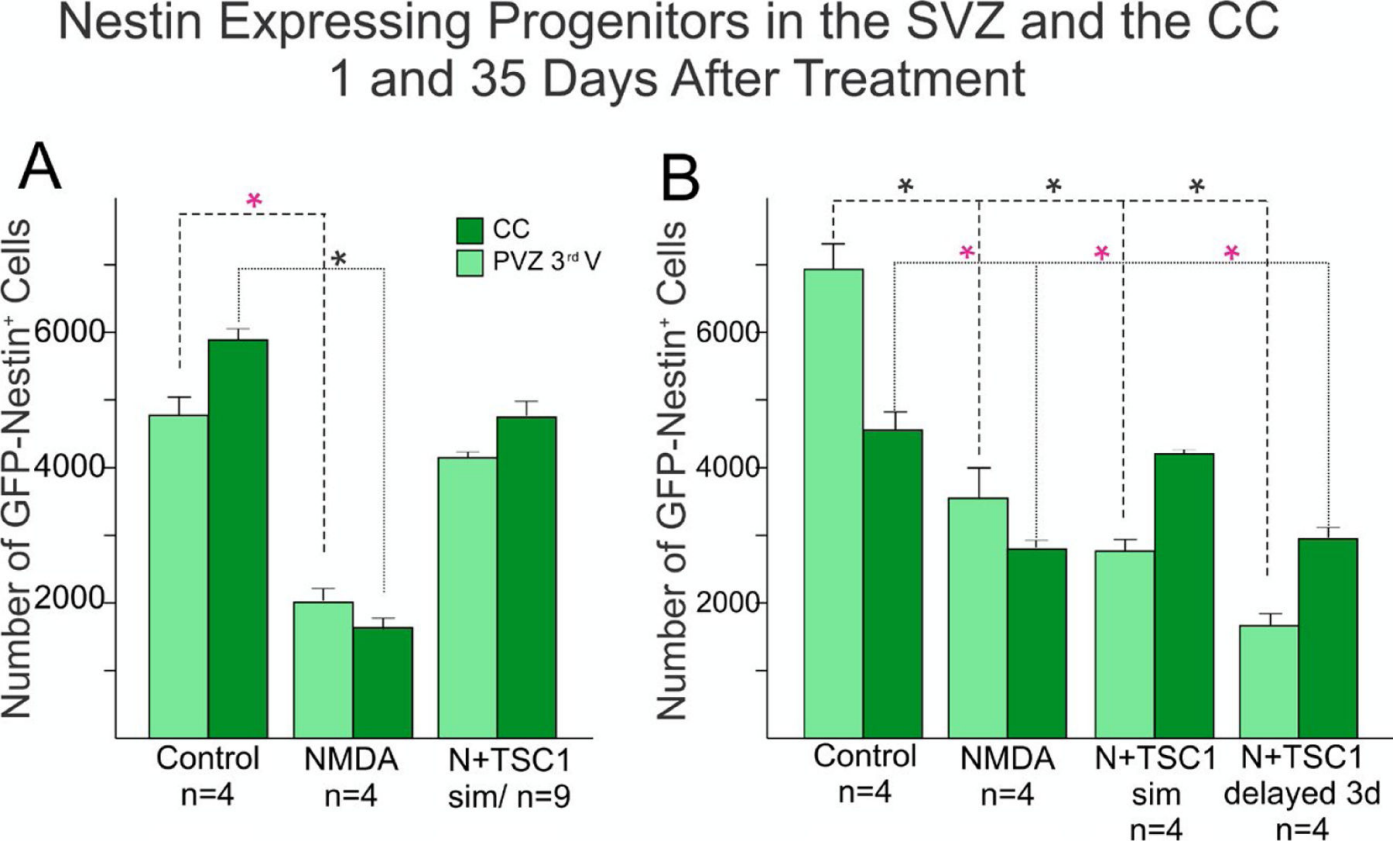
Comparison of the total number of Nestin-GFP expressing progenitors in the SVZ of the third ventricle and the CC. Sagittal sections were examined, nestin-expressing progenitors were counted using the Optical Fractionator Probe (mbf) and the 63X oil objective. A) The total number of nestin-expressing progenitors was extremely reduced in NMDA injected mice as early as 1 day after GME with more cells left in the SVZ than those that migrated to the CC. Mice that received the injection of NMDA + TSC1 simultaneously showed a 25% nestin-expressing cell loss as opposed to almost 66% reduction found in mice injected with NMDA alone. The differences were significant between saline and NMDA injected mice and non-significant between saline or untreated controls, and N + TSC1 mice indicating that the number of nestin-expressing cells both in the SVZ and CC was considerably close to controls when compared to mice injected with NMDA alone. Significance for the SVZ(*P)<0.05, and for the CC (*P)<0.05 versus their respective control. B) Thirty-five days after injection there were still more nestin-expressing cells in mice receiving saline injections than in those injected with NMDA in the presence or absence of TSC1; with NMDA alone there was a reduced loss of the total number of nestin-expressing cells from 66% to 45%. At this time point NMDA+TSC1 injected simultaneously reflected a total reduction of 39% nestin-expressing cells. A more pronounced reduction was seen when TSC1 injection was delayed 3 days after NMDA administration. Nonetheless, more cells had migrated to the CC than in NMDA injected mice. Bars represent the means ± SE for each group.*P<0.05 versus respective control. The difference in cell numbers found in the CC between N+TSC1sim and N + TSC1 administered 3 days later, was significant (*P<0.01), suggesting that the sooner we inject TSC1, the more nestin-expressing cells will be spared.

We next compared across treatments the number of nestin-expressing cells in the SVZ and the corpus callosum (CC) using the Optical Fractionator Probe (mbf). There was a considerable loss of nestin-GFP progenitors after NMDA injection when compared with mice injected with saline (Figure 4). One day after injection the total number of nestin positive cells had been reduced 66% of what was present in saline injected mice size the vast majority of nestin-expressing cells appeared to have been spared reflecting a loss of 16%. In the saline injected group the distribution of cells was almost equal between SVZ and CC, while in NMDA injected mice there were more cells left in the SVZ than in the CC perhaps due to a decreased cell migration (Figure 4A). Thirty-five days after treatment, more nestin-expressing cells were present in the CC in mice receiving NMDA + TSC1 simultaneously accounting for 39%, while in mice where the TSC1 treatment was delayed 3 days, the number of cells lost accounted for 59% with respect to control mice, while the reduction in NMDA treated mice was 45%. Overall the total number of these cells from the two regions combined was lower than in mice that received NMDA + TSC1 simultaneously. At this time point in control mice and those treated with NMDA + TSC1 displayed more GFP labeled cells in the CC than in the SVZ suggesting that migration of nestin-expressing cells towards the CC had occurred timely one day after injection. By thirty-five days after treatment mice that received NMDA regardless of the presence or absence of TSC1, showed a reduction on the total number of nestin-expressing cells in both regions when compared to control brains. Interestingly, both groups receiving N + TSC1, simultaneously or delayed, displayed more nestin-expressing cells in the CC than in the SVZ at this time point (35 days post-injection “35dPI”, Figure 4B). Several reasons exist for these findings that are discussed in the next section.

### Regeneration of the nestin-expressing cell population via mobilization and proliferation

**Figure 5.**
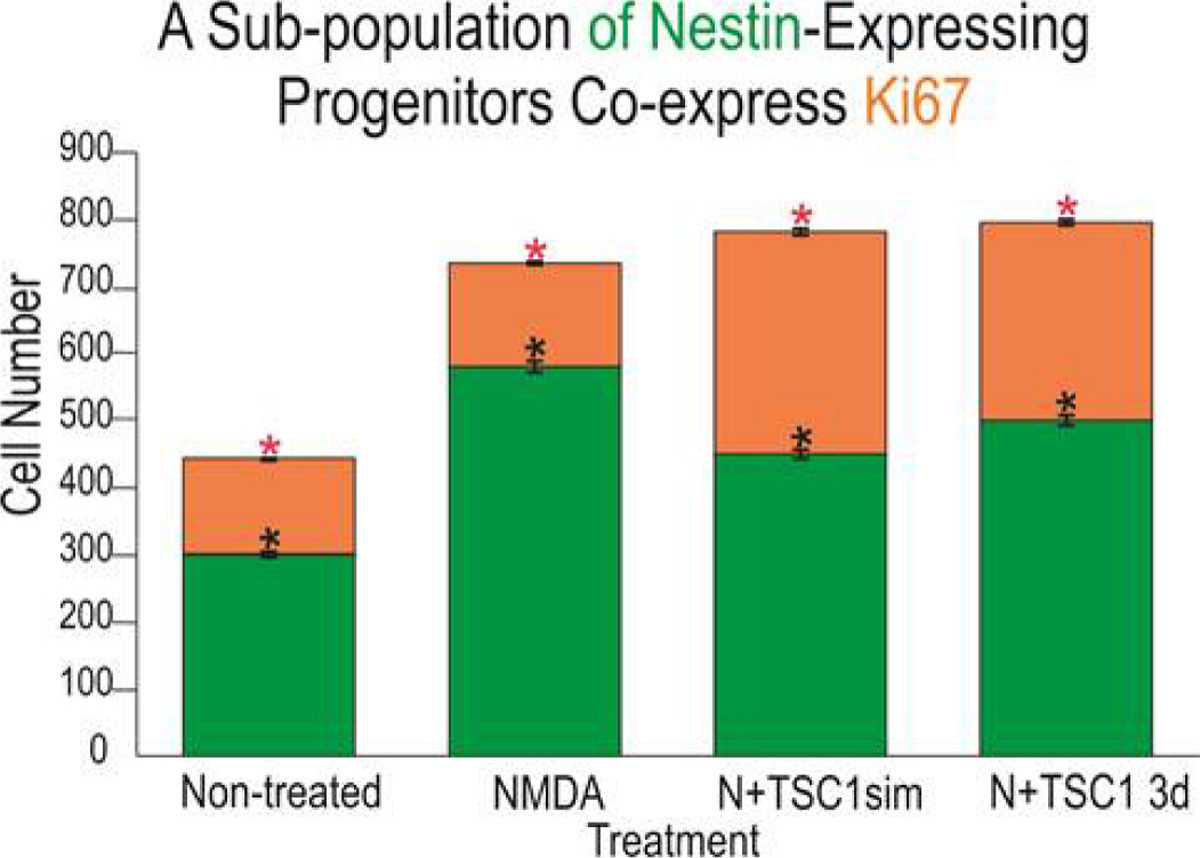
The subventricular zone regenerates *via* proliferation of a sub-population of nestin-expressing progenitors as well as *via* direct mobilization of quiescent nestin-expressing cells. Seven days post-injection (PI) ten fields were selected around the SVZ of the third ventricle in the ipsilateral hemisphere in sagittal sections. First, only cells expressing nestin-GFP were counted (green portion of the bars), these cells were negative for the proliferation marker Ki67. Values are expressed as mean ± SEM; *p<0.01 across treatments versus control. Interestingly, in mice treated with NMDA followed by a 3-day delayed TSC1 injection, the total number of nestin positive cells was slightly but significantly greater than in NMDA treated mice. The number of nestin+ cells in each experimental condition was compared with that of the non-treated mice. The co-expression of Ki67 and nestin in the SVZ (orange portion of the bars) was also assessed by counting double labeled cells in the same ten fields around the SVZ. The total number of nestin-expressing cells in the control group was lower than in the brain of treated groups. Approximately, one third of these cells co-expressed nestin and Ki67. NMDA treated mice also showed new nestin-expressing cells generated *via* cell proliferation as assessed by ki67 expression. A striking finding was that in mice that received NMDA +TSC1 either simultaneously or three days after NMDA administration, twice as many nestin-expressing cells had formed *via* cell proliferation with respect to the control group. In the N+TSC1 injected simultaneously the total number of nestin-expressing cells was almost doubled from which nearly 50% were new progenitors generated *via* cell proliferation. In the case where NMDA was injected and TSC1 treatment was administered 3 days later, the number of non-proliferative nestin positive cells was slightly larger while those generated *via* proliferation amounted to around 40%. Values are expressed as mean ± SEM; *p<0.01 across treatments versus control (non-treated).

In order to understand if nestin-expressing cells in the SVZ were being recruited by TSC1 solely by mobilization from a quiescent state into an active migratory state, we examined the total number of ki67 positive cells that were not either nestin or CNPase positive as shown in Figure 5. Only a small fraction of cells was found to be ki67 and unrecognized by the OL marker CNPase or the progenitor marker nestin. Interestingly, at this time point the number of nestin-expressing cells remained virtually equal across treatments and there were only a few CNPase expressing cells in this region even when mice received NMDA+TSC1 simultaneously as shown in (Figure 5). Nonetheless, a more robust number of CNPase positive cells was found suggesting maturation of OL induced by TSC1 *in situ*. Next in order to demonstrate proliferation of nestin-expressing cells, we searched for a nestin-GFP+/Ki67+ cell population. Immunocytochemistry for Ki67 (a cell proliferation marker) and nestin revealed that a fraction of nestin-expressing cells was generated by cell proliferation. The total number of cells expressing Ki67 was counted. Seven days after the intraparenchymal injection of either saline or NMDA alone resulted in very similarly low numbers of nestin-expressing cells that were co-labeled with anti- ki67. The two groups injected with NMDA and treated with TSC1 simultaneously or three days later displayed virtually twice as many nestin-expressing cells with respect to the saline control. These results confirm that a pool of nestin-expressing progenitors is generated via cell proliferation (Figure 5).

### *Ex-vivo* experiments

**Figure 6.**
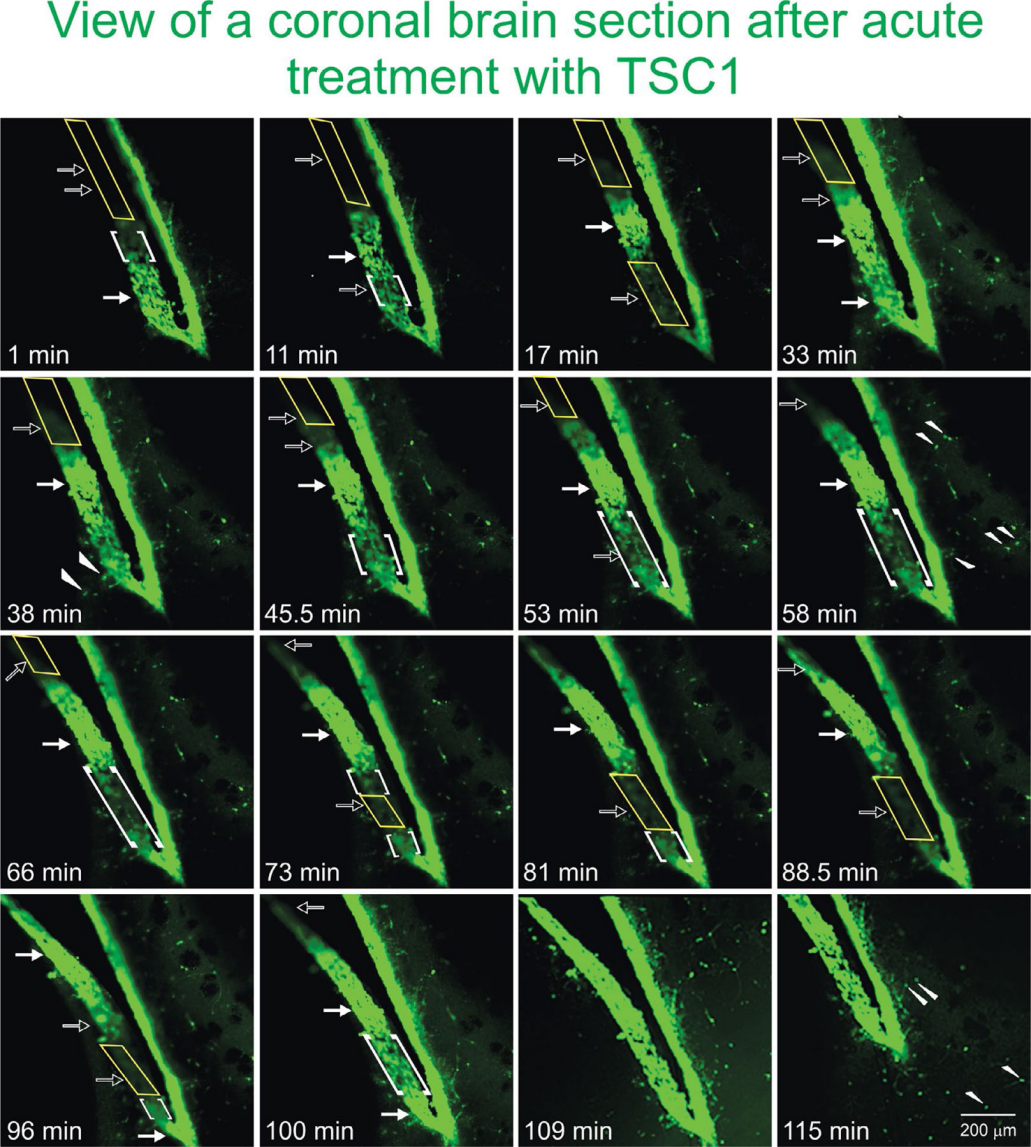
TSC1 elicits the “awakening” of nestin-expressing cells in the SVZ of the lateral ventricle. To assess the ‘awakening” and migration of NSC, we used fresh 300 μm thickness brain slices from the nestin-green fluorescent protein (GFP) mouse at 7 months of age to be able to distinguish *di novo* GFP-nestin expression. Views of the lateral ventricle from a coronal brain section treated with TSC1 show a single plane of focus during 115 min. Note a pronounced thickening of the ventricular wall and the subventricular region within minutes of exposure to TSC1. We observed a dynamic behavior of GFP-nestin-expressing cells. Some regions that were negative (yellow rectangles), became intensely labeled for nestin-GFP at time 1 min post treatment, gradually becoming visible starting at 11 min (open arrows) and continued showing the 100% of the SVZ light by intense GFP fluorescence by min 109 (1h 49 min, solid arrows). At the same time, zones of the SVZ that expressed intense to moderate green fluorescence at time 0 became gradually negative until no GFP was detected by minute 88 (1h 28 min). Interestingly, the same region started to light up again by min 100 and became intensely labeled 9 min later, time at which the full view of the lateral ventricle was GFP positive. Concomitant to the development of these phenomena, single bipolar cells were seen to migrate from the SVZ into the adjacent parenchyma (arrowheads). Initially, just a few cells were visible but with time rows of cells perpendicular to the SVZ were observed (small arrows). Note that 11 minutes after addition of TSC1, a nestin negative area became positive while the opposite occurred below that region. After 17 min the same area had turned off and turned green again by 33 min after TSC1 was added to the tissue slice. Starting at 17 min green cells appeared to have moved beyond the SVZ into the brain parenchyma. This region of the SVZ was the most active as it went gradually on (at 33 and 100 min) and off (at 17 and 73min). The complete SVZ became nestin-GFP positive at 109 min. Migrating cells had increased in number with time and of the SVZ took place during the first 33 min.

To confirm the direct recruitment of quiescent nestin-expressing progenitors we examined the effects of TSC1 on the emergence and migration of nestin-expressing cells in the adult brain using seven month-old mouse brain. Sections were placed in culture medium under an inverted microscope equipped with camera for time lapse experiments. When the time lapse was started and TSC1 was added, it was considered to be time “0”. GFP-nestin expression was visible from the beginning and remained sustained in the SVZ 1h after treatment. Nonetheless, during this time we observed areas of the SVZ that were nestin negative and with time the darkness of this region became intensely labeled with green fluorescence. While another region that was intensely green at time 0 was slowly becoming dark around 50 min after the TSC1 treatment. Interestingly, a single treatment with TSC1 elicited several waves of nestin-expressing emerging cells. Single cells were also seen; some appeared to migrate away from the densely populated SVZ into the adjacent parenchyma remaining green for at least 1.45h (115min, duration of the experiment). We observed that at time 0 the SVZ was narrower than at later time points. Twenty-four hours after treatment, the intensity and extent of GFP-nestin expression had declined but did not disappear (Figure 6).

### Astrocytes response to WMI

**Figure 7.**
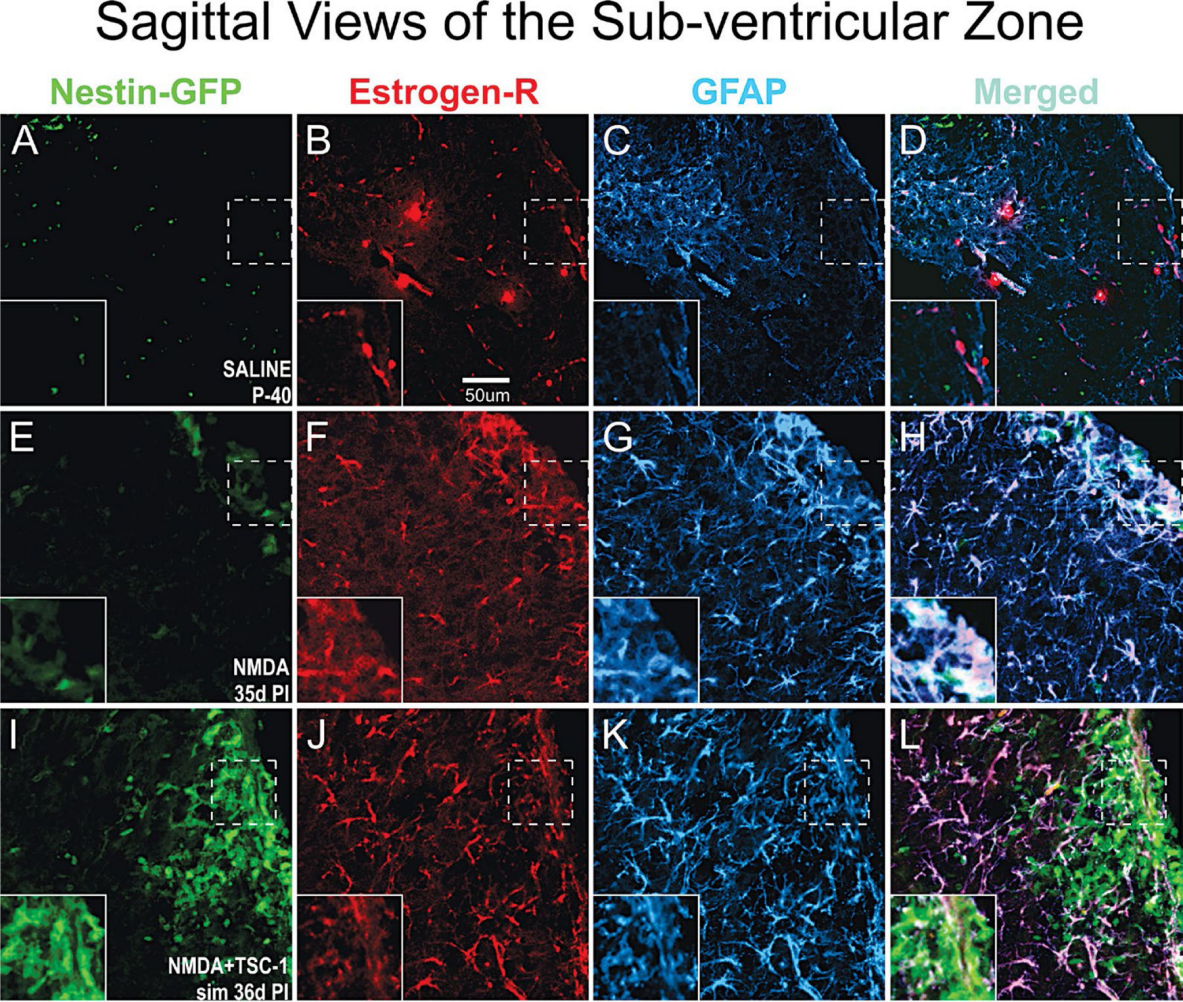
In the SVZ Estrogen-R shifts localization from the vasculature to astrocytes upon GME. Double immunofluorescence for estrogen receptor alpha (Estrogen-Rα) and glial fibrillary acidic protein (GFAP). A-D Saline treated mice at P40 the estrogen-Rα was present only in blood vessels but not in astrocytes. In contrast, in NMDA recipients 35d PI (d = days; PI = post injection) estrogen R colocalized with GFAP mainly in the cell body of astrocytes and it was also intensely expressed in blood vessels. Astrocytes were reactive as they were enlarged and fibrous with prominent processes strongly expressing GFAP (E-H). Interestingly, in NMDA+TSC-1 treated mice (I,J,K,L) most but not all astrocytes were still enlarged and their cell body prominently labeled with GFAP. Other astrocytes showed smaller body and processes, they seemed non-reactive and in smaller numbers only a few astrocytes co-expressed ER-α. The label was diffusely distributed along fibers rather than in the astrocytes cell bodies. Estrogen-R was not expressed by nestin-GFP progenitors (inserts).

We next examined the status of astrocytes 35 days after NMDA injection alone or with TSC1 in the sub-ventricular zone (SVZ). To identify astrocytes we examined GFAP expression that allowed us to visualize their status (*i.e*., normal astrocytes or reactive ones). We also determined the expression of the estrogen-R-α antibody. Nestin-expressing cells did not express estrogen receptor in this model of premature birth. In the SVZ, astrocytes were of normal appearance in mice injected with saline, and estrogen-R-α was expressed by some blood vessels only (Figure 7A to D). In contrast, after NMDA injection, in the SVZ and in the adjacent parenchyma astrocytes showed more prominent cell processes intensely expressing GFAP. Astrocytes responded to GME with the up-regulation of estrogen receptor-α which was also expressed in some blood vessels (Figure 7 E–F).. After the injection of NMDA+TSC1 astrocytes did not appear reactive, they appeared to be in smaller numbers and only a few astrocytes co-expressed GFAP and estrogen-R. The estrogen-R label was diffusely distributed along fibers rather than in their cell bodies. The estrogen-R was not expressed by nestin-GFP progenitors (Figure I-L). After the NMDA+TSC1 intervention astrogliosis was still present and most astrocytes co-expressed Estrogen R- and GFAP (Figure 7 J,K). Nestin-expressing cells did not display ER-α.

**Figure 8.**
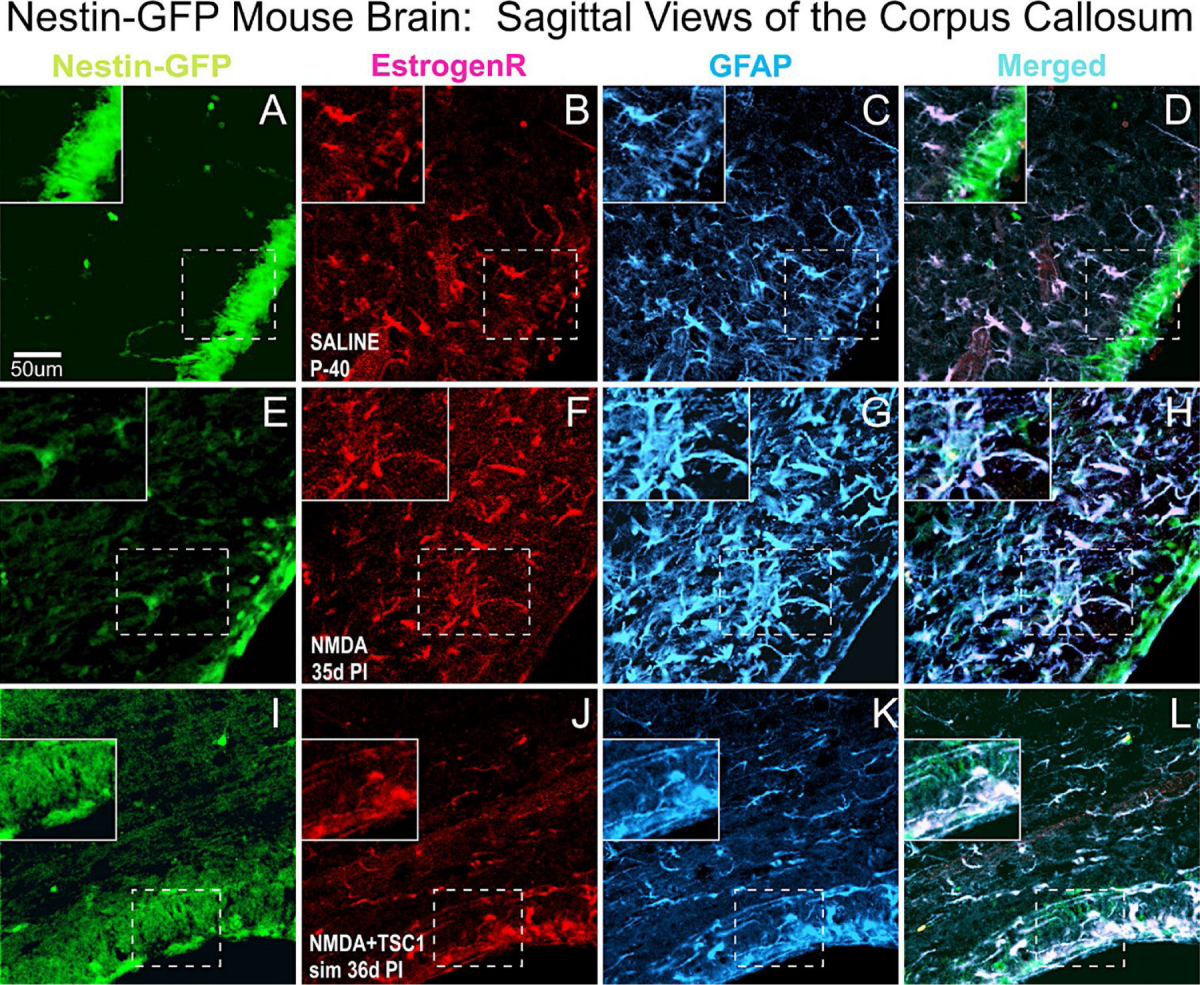
Sagittal Views of the Corpus Callosum. Double immunofluorescence for estrogen receptor (ER-α) and glial fibrillary acidic protein (GFAP). The corpus callosum (CC) showed that in non-treated animals (A-D) ER-α was present mainly in blood vessels and in a few astrocytes. In contrast, in NMDA injected mice (E-F) ER-α colocalized with GFAP mainly in the cell body and it was intensely expressed in blood vessels as well. Astrocytes were reactive, they were intensely labeled and with a fibrous aspect. Interestingly, in NMDA+TSC-1 treated mice (H,I,J,K), the astrocytes seemed non-reactive, they were less numerous and their cell bodies were small as well as their GFAP labeled cell processes and they were present in smaller numbers and only a few of them co-expressed ERα. The ERα was almost not seen in blood vessels and those that expressed displayed a diffusely distributed ERα and GFAP along their fibers rather than in their cell bodies. ERα was not expressed by nestin-GFP progenitors. Inserts show the absence of ERα label in nestin-expressing cells adjacent to those GFAP/ERα co-labeled cells.

We also evaluated the status of astrocytes 35 days after NMDA injection alone or with TSC1 in the CC. The corpus callosum (CC) showed that in P40 non-treated animals (A-D) ER-α was present mainly in blood vessels and in a few astrocytes. In this region NMDA seemed to impact much stronger astrocytes that were very reactive Figure 8G. This strong astrogliosis was accompanied by the shifting on the expression of ER-α from blood vessels into astrocytes and both markers co-localized in most astrocytes (Fand H). In contrast in mice co-injected with NMDA+TSC1(Figure 8 I–L) estrogen receptor immunoreactivity had considerably decreased, as well as the astrogliosis suggesting a normalization of the microenvironment.

### Caspase 9 expression is upregulated by NMDA and attenuated by TSC1

**Figure 9.**
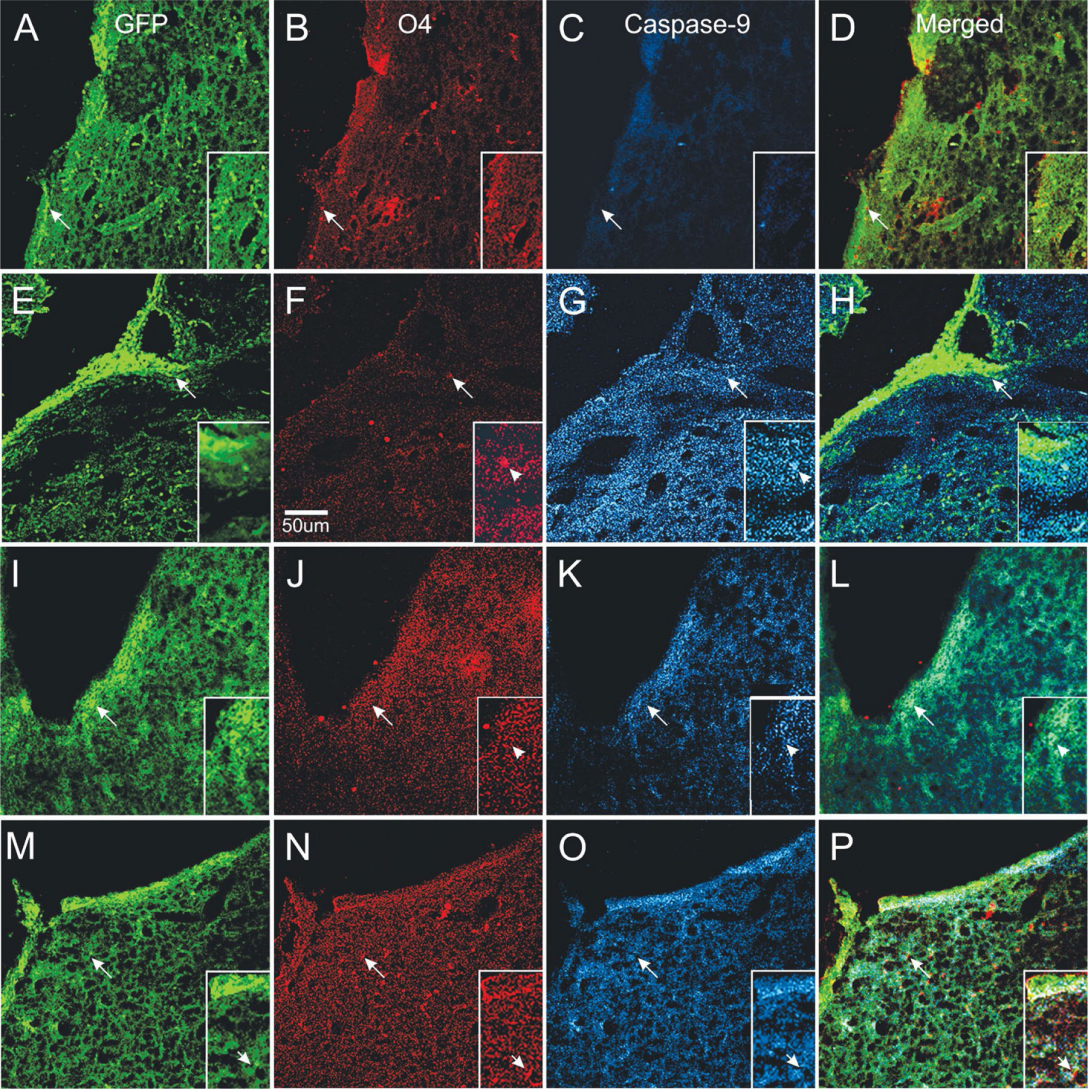
Differential neuroprotection produced by TSC1 administered during or after excitotoxicity. Control mice injected with saline (A–D). A). Several nestin positive cells were located in SVZ and in some areas of the ventricular wall. B) O4 expressing cells were also present in the SVZ and the adjacent parenchyma. C) Caspase 9 was not expressed in saline injected mice. D) Merged views of the three markers. Mice treated with NMDA alone (E–H) showed intensely nestin labeled progenitors at the wall of the ventricle (E) =creo que no debe llevar el parenthesis izquierdo esta E=almost none in the SVZ and numerous single cells in the neighboring parenchyma. F) Very few O4 positive cells could be observed with hairy cell processes. G) These O4 positive cells co-expressed caspase 9. Single cells with fewer processes were also seen. In addition, the full area surrounding the lateral ventricle of the injected hemisphere showed caspase intense staining as “puncta”. These puncta seemed to be predominant in comparison to the extent of O4 labeled puncta. H) Very few nestin positive cells co-expressed caspase 9. When NMDA was injected simultaneously with TSC1 (I–L), nestin-labeled cells were seen across the wall of the ventricle, SVZ and adjacent parenchyma (I). There was much more O4 expressed in small size OLPs; some could be seen as bipolar cells with long processes than in mice injected with NMDA alone. More O4 labeled puncta were observed (J). In these mice, some but not all, nestin-expressing cells co-expressed caspase 9 (L). The delayed administration of TSC1 3 days after NMDA injection resulted in a spongy-like tissue M–P). Nonetheless, both the expression of nestin (M) and O4 (N) were preserved and O4 positive cells were negative for caspase 9 (O). It also appeared as if caspase 9 was labeling cells other than nestin-expressing and O4 labeled cells, in which cases caspase 9 seemed to label flatter cells (O).

To determine which progenitors were more at risk of dying after excitotoxicity, we examined the expression of caspase 9 with respect to either nestin or O4 expressing cells. Caspase 9 was not expressed in mice injected with saline. In contrast, NMDA injection resulted in a strong caspase 9 expression and obliteration of O4 immunoreactivity. Caspase 9 was expressed in cells and also in a pattern of “puncta” scattered along the SVZ and neighboring parenchyma. In some cases caspase 9 and nestin colocalized but the vast majority of nestin-expressing cells were negative for caspase. Mice injected with NMDA + TSC1 simultaneously were in much better condition, O4 was expressed in the SVZ in the OL cell body as well as in their cell processes. Caspase 9 expression was reduced and confined mainly to the SVZ. The effect of the NMDA injection followed by TSC1 treatment 3 days later were similar in that O4 was expressed in parenchyma adjacent to the SVZ and caspase was expressed in a few regions of the SVZ with moderate intensity. The main difference between the simultaneous and the delayed treatment was that OL displayed O4 hairy cell processes while brains that received TSC1, 3 days after NMDA displayed rows of small O4 expressing cells perpendicular to the wall of the ventricle and a thinner layer of nestin positive cells in the SVZ. Caspase appeared to overlap with nestin in a few cells but, not with O4 (Figure 9).

### Open field assessments

**Figure 10.**
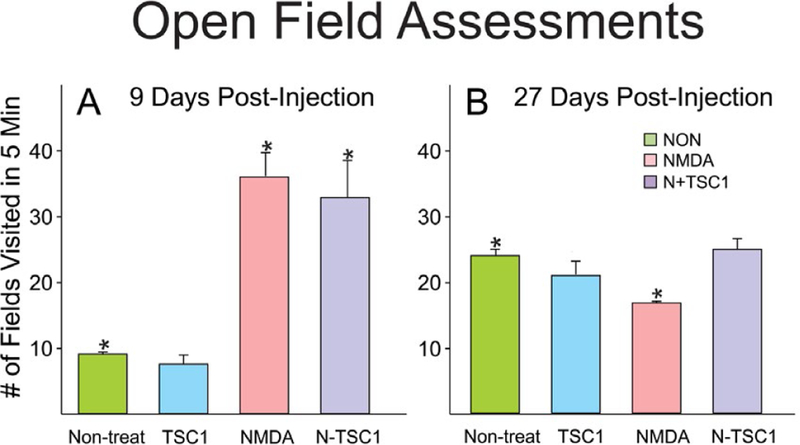
NMDA induced hyperactive behavior at early time points. Initially, non-treated mice and those that received TSC1 injection 9 days earlier had comparable behavior and the small differences were not significant. In contrast, animals injected with NMDA alone or NMDA + TSC1 were characterized by hyperactivity and the difference was significant with respect to control (non-treated) mice). Twenty-seven days after treatment, the behavior of all groups was similar yet the difference was significant for NMDA-treated mice when comparing it to that of non-treated mice. In contrast, the behavior of mice in groups non-treated, TSC1 and N-TSC1 was equivalent and the differences were nonsignificant anymore.

Animals were evaluated for open field behavior. The open field test was used to measure locomotor activity and exploratory drive. The arena was indirectly illuminated and consisted of a box 28 × 28 x divided in 4 quadrants. Mice were handled by the base of their tails at all times. Mice were placed into the center, of the open field and allowed to explore the apparatus for 5 minutes. After the 5-minute test, mice were returned in their home cages. NMDA administered at P4 gave rise to induced locomotor hyperactivity assessed by open field tests starting 9 days after treatment, while non-treated mice and those receiving TSC1 alone displayed a much lower number of fields visited in 5 minutes. With time after injection these differences tended to disappear. Twenty-seven days after treatment there were no significant differences as shown in Figure 10.

## Discussion

Promotion of myelination in the CNS as a potential strategy for therapeutic intervention to overcome myelin deficiency is not a new concept. In 1996, Mc Morris and McKinnon [39] reviewed and compiled data from various laboratories including their own work where it became clear that OL development and maintenance are regulated by growth factors. Here we would like to expand the category of OL regulators from “growth factors” to both, “growth and trophic factors” from which IGF-1 was used on EAE animal models. These mice showed thin myelin around axons [40,41] with an improvement on clinical symptoms. Many aspects on the effects of IGF-1 on OL and myelination/remyelination have been studied. Yet, a cure for myelin deficiency remains elusive. In the current paper we focused only on an intervention that would benefit premature neonates by regenerating their forming white matter after excitotoxicity.

### TSC1 neutralizes the vulnerability of nestin-expressing progenitors to GME *in vivo*

During development, intrinsic genomic mechanisms of commitment and lineage restriction are modulated by environmental signals in embryonic cells. This has become more evident with studies of neural cells in culture and neural cell transplants that have been very instrumental in the assessment of the potential of cell grafted cells for CNS repair [42]. The advent of stem cell biology offers a more promising future for cell replacement therapies in dys- and de-myelinating diseases. There is an increasing number of studies dealing with stem cells preparation, and the successful generation of OL in large and reproducible numbers [43,44]. Based upon BrdU incorporation, we previously found that in the unaffected and myelin deficient rat mutant (md rat), quiescent stem/progenitor cells are stimulated to proliferate by TSC1 [36]. In the present study using the same animal model, we sought to determine how nestin-expressing progenitors responded to NMDA injections and the effects TSC1 would produce on the nestin-expressing cell population. The NMDA injection resulted in a total loss of nestin-expressing cells in the ventricular wall and the SVZ, both, the SVZ and brain parenchyma presented a spongy appearance. In contrast, in the presence of TSC1 nestin labeled cells were found at the wall of the ventricle, as well as in the SVZ. These cells appeared to migrate into the parenchyma as if NMDA had not disturbed their known path and abilities. Thus, TSC1 made the environment permissive for survival of nestin-expressing cells. The stereology data showed that one day injection 66% of the nestin-expressing cell population had been lost due to excitotoxicity caused by NMDA while TSC1 seemed to protect nestin-expressing cells from NMDA when injected simultaneously, having lost only 16% of the nestin labeled cell population. At this early time point, the number of cells found at the ventricular wall together with those in the SVZ and cells found in the CC was very similar within their own group (*i.e*., NMDA group), indicating that some nestin-expressing cells had already migrated towards the CC prior to the insult. By 14 days the total numbers of GFP-nestin positive cells across treatments with or without TSC1 were very similar and represented 50% of the total population in non-treated and saline injected litter mates. At PI day 1 there were many more nestin positive cells in mice treated with TSC1 but were not anymore present 13 days later; this could be due to either that some of these cells had lost nestin expression in the SVZ while they were migrating and reached the CC. This is plausible because we have previously shown in a rat model of inherited leukodystrophy that OLPs respond to TSC1 by moving forward from a progenitor stage to a pre-myelinating OL [36]. What we learned from this part of the current study is that when NMDA and TSC1 were injected simultaneously, the GFP population was largely protected and that at later time points, such as 35 days after treatment, there seems to be a second wave of nestin positive cells arriving to the CC either because they had a delayed migration or because they were progenies of the original nestin-expressing progenitors that survived the insult. After the delayed injection of TSC1 the number of nestin-expressing cells was equivalent to that seen at PI-14. This could be due to the fact that the delay in administering TSC1 led to a delayed proliferation of nestin-expressing cells present after the excitotoxic insult. Alternatively, the TSC1 injection could have enhanced the migration of pre-existing and new nestin positive cells beyond the SVZ. A third possibility is that some cells stopped expressing nestin as they would tend to start expressing early markers for a specific cell type, most likely OLPs, as we have previously shown [36].

It has been reported that as OLPs move towards a more mature phenotype, they become more vulnerable to oxidative stress that leads to induced cell death [45], therefore, we next examined the presence of the stress protein HSP-32 as a marker of oxidative stress across treatments. Non-treated mice did not express HSP-32 because it was (yo le pondría “they were”) uninjured. Mice treated with NMDA alone failed to display HSP-32 expression. While, in mice treated with NMDA+TSC1 nestin-expressing cells co-expressed HSP-32 at the wall of the ventricle and in the SVZ. Nestin negative/HSP-32 positive cells were frequently found migrating along nestin radial cell processes. HSP-32 expression indicated that newly formed cells responded to WMI but most likely would be rescued by HSP-32. In contrast, the inability of neural progenitors to express HSP-32 in mice that received NMDA only led to massive cell loss and lack of tissue rescuing and regeneration. Therefore, the more cells expressing HSP-32, the more promising appeared to be their survival and overall brain recovery.

### TSC1 is a neuroprotector and regulator of neurogenesis in WMI

Based upon BrdU incorporation we have previously shown that TSC1 mobilizes and triggers the proliferation of nestin-expressing progenitors in a rat model of leukodystrophy [36]. More recently we have shown in the same mouse model of excitotoxicity used here, that a fraction of CNPase positive OL co-expressed Ki67, indicating that TSC1 generated some OL via OLPs proliferation. Moreover, numerous cells had proliferated as determined by ki67 expression and the number of these cells was larger than those co-expressing ki67 and CNPase. Those higher numbers were seen at early time points after TSC1 injection decreasing in function of time [38]. Here using the proliferation marker Ki67 we sought to ascertain if nestin-expressing progenitors had proliferated. Indeed we found that at early time points (24h after the injection) numerous nestin-expressing cells were labeled with anti-Ki67. The most remarkable finding was that when TSC1 was administered three days after NMDA, the treatment gave rise to twice as many cells expressing both, nestin and Ki67, as well as 1.7 more Ki67/CNPase expressing cells with respect to non TSC1, NMDA injected mice.

The *ex-vivo* experiments were very useful to demonstrate that when brain tissues enter in contact with TSC1, the expression of nestin-GFP in the ventricular/subventricular zone (SVZ) is rapidly elicited and it increases fast, even in adult mice. Moreover, the effect is sustained and elicits waves of nestin-positive cells that emerged not only immediately after treatment but, in a delayed manner, in different regions of the SVZ. This fact is very promising not only to address perinatal white mater regeneration, but to replace cells lost in neurodegenerative disorders by expansion of self-neural progenitors and further direction of their phenotype fate. Moreover, these results show that the recruitment of nestin-expressing progenitors involves two subpopulations, new cells generated via proliferation and non-proliferative cells that respond to TSC1 by expressing nestin and migrate into the parenchyma. Both of these populations appear to contribute to the regeneration of the CNPase-expressing population.

### Other mechanisms for neuroprotection by IGF-1

It is known that estrogen actions are mediated mainly by nuclear estrogen receptors ERα and ERβ, additionally nongenomic membrane effects have also been reported [46]. ER receptors become activated after binding to their ligands [47,48]. Both receptors temporarily may be expressed by a given cell [49,50] and might work either synergistically or antagonistically, depending on the cell/tissue. ERs actions seem to depend on differences in transcription factors inherent to a specific tissue [51,52] and [53] using estrogen receptor knock-out (KO) mice have shown ERα protective effect of estrogen treatment. Moreover, ERα expression is necessary in astrocytes, but not neurons, to prevent macrophage and T-Cell inflammation in the CNS. It has also been shown [54] that treatment with an ERα ligand is highly selective *in vivo*, mediating both anti-inflammatory and neuroprotective effects in a mouse model of MS. Therefore, we wanted to determine if TSC1 could be mediating its neuroprotective action at least in part by acting on astrocytes.

Caspases regulate the maturation and release of cytokines. The apoptotic caspases (caspases 3, 6, 7, 8 and 9) execute apoptotic cell death through extrinsic or intrinsic pathways [55]. In neurological disease, inflammatory caspases promote immune activation, whereas apoptotic caspases are activated in neurons in response to immune molecule-mediated cytotoxicity, decreased growth factor signaling and excitotoxicity [56,57]. Caspases 3, 7 and 9 are primarily known for mediating apoptosis, but they might also mediate activation of microglia in response to lipopolysaccharide [58,59]. The inflammatory response involves the recently discovered multiprotein complexes termed ‘*inflammasomes*’ [60]. An inflammasome is a cytosolic, multiprotein platform that enables the activation of pro-inflammatory caspases and a potent inflammatory response [61]. Inflammasome activation has been implicated in the mechanisms underlying infectious, immune and inflammatory processes [62]. In the current study caspase 9 was expressed mainly in O4 expressing cells even 35 days after treatment with NMDA with or without TSC1 suggesting that after excitotoxicity there is a prolonged mild inflammatory process to which the O4 OL population responds still 35 days after the excitotoxic insult. These results expand on the previously reported findings on the vulnerability to oxidative stress that leads OL and in particular O4 positive cells to induced cell death [45].

Recently, it has been reported that mature IGF-1 triggers three enzymes, mitogen-activated protein kinase (MAPK), phosphatidylinositol 3-kinase (PI3K), and phosphoinositide phospholipase C-γ (PLC-γ), which are its predominant downstream regulators [63]. It also decreases autophagy induced by NMDA in cultured hippocampal neurons. The protective effect of IGF-1 against autophagy was accompanied with up-regulation of phospho-AKT (p-AKT) and phospho-mTOR (p-mTOR) [63]. Our data from the Caspase-9 immunofluorescence revealed an extensive distribution of puncta in what appeared to be labeling intracellular, cytosolic structures in O4 expressing cells. Moreover, these puncta were also abundant in extracellular regions suggestive of being localized in the myelin. Another indication of extended inflammation is the expression of estrogen in astrocytes rather than only in blood vessels.

### NMDA induced hyperactivity that was not mitigated by TSC1 at early time points

The NMDAR plays a critical role in functional and structural synaptic plasticity [64]. Nonetheless, it is not clear how NMDA would impact this receptor in glial cells. Mathis *et al.* have reported the induction of seizures by NMDA [65]. Yet, low doses of NMDA produce epileptic discharges that may occur without clinical manifestation but, it is known that cell death occurs and hyperactivity might be the result from neuronal death. Initially we expected that TSC1 would mitigate hyperactivity but the NMDA effects predominated at early time points. An explanation for these surprising results may come from a report on glutamate acting on NMDA receptors by decreasing IGF-1 receptor tyrosine phosphorylation that reduces the survival signaling in cultured neurons [66]. Another contributing factor could be the delay in OLPs migration as we previously reported using the same mouse model [38]. This delay of newly formed OLs reaching naked axons might also contribute to the hyperactivity due to delayed myelination. At later time points OL had myelinated axons and therefore, neuronal function may have normalized.

Previous results generated by Dodge *et al.* showed that delivering IGF-1 to the CNS is sufficient to delay disease progression in a mouse model of familial ALS and demonstrated for the first time that IGF-1 attenuates the pathological activity of non-neuronal cells that contribute to disease progression [67]. In the 1990’s recombinant form of IGF-1 (peptide) was used in two ALS clinical trials by Cephalon. They administered the molecule by subcutaneous injection daily and showed modest effects without improving survival. Another clinical trial confirmed that IGF1 did not slow either the progression of weakness nor functional deterioration in people with ALS (A. Madsen, MDA/ALS Newsmagazine). Seen in retrospect most likely, these trials did not show robust results due to three main factors: A) its delivery method because most of it did not reach the CNS; B) its short half-life; and C) the most important is the nature of the disease to be treated. ALS is a progressive neurodegenerative condition that implies a treatment that will need to be administered either continuously or periodically to protect cells and to support functional cells with a concomitant delay on disease progression. With the current technology available gene therapy offers a more stable and constant “production” and “delivery” of IGF1 to treat a neurodegenerative disease like ALS. In contrast, here we demonstrate that TSC1 is a valid and efficient formula to mitigate white matter damage in the premature neonate. We previously showed that separately both transferrin and IGF1 support OL survival, maturation and increase myelination by both normal and myelin deficient OL [14,36,68]. Thus, it is not surprising that a single administration of the cocktail is sufficient to trigger and generate a second wave of progenitors to form the white matter. Therefore, in order to overcome the extensive tissue loss typical of PVL, the intervention should occur as early as possible. One can predict that a continuous or periodical delivery of TSC1 during the perinatal life would be deleterious as it would alter the environment and OLPs will not know if they have to move forward in the lineage or remain immature and produce more OL if the environmental conditions allow for it. In the adequate milieu after the excitotoxic insult and a single TSC1 dosis, OL will mature and synthesize all myelin components and myelin.

### Some of the mechanisms that may be modulated by TSC1 include iron homeostasis, neural cell survival and apoptosis

Various trophic factors have shown promise for neuroprotection, but given their role in normal development, their effects as therapeutic agents have not been easy to elucidate. IGF-1 is important for growth and maturation of the fetal brain, as well as for differentiation of OL precursors [69)]. After insult to the CNS, IGF-1 has “pro-“survival properties that help prevent or attenuate the consequences of injury to the perinatal CNS from hypoxia and excitotoxicity [70–72]. Moreover, it has also been proven effective after intranasal administration [73].

There are various signaling pathways involved in IGF-1 normal activity in the CNS. Ligand binding to IGF-1 receptors (IGF1Rs) triggers receptor autophosphorylation and association with docking proteins. Cells trigger apoptosome formation by the release of cytochrome c from damaged mitochondria. The cytochrome binds to ATP-dependent proteolysis factor 1(Apf-1) monomeres which then create APF heptameres or heptamere apoptosomes. Pro-caspase-9, is recruited either in the apoptosome or by binding directly to Apf1, this activates caspase 9, cleaves pro-caspase-3 and activates it. Caspase-3 in turn breaks down cellular components for their recycling [74]. Thus, in our experimental paradigm exogenous IGF-1 contained in TSC1 may prevent mitochondrial damage and cytochrome release, blocking the caspase chain of events that lead to apoptosis. Hypoxia-inducible factor 1α (HIF-1α) activation is a key modulator of the protection against subsequent HI injury that is induced by hypoxic preconditioning [75–76]. HIF-1α is a neuronal transcription factor that stabilizes during hypoxia by binding to HIF-1β. Following stabilization, it produces a variety of downstream targets that are neuroprotective, including IGF-1, vascular endothelial growth factor, and erythropoietin [77].

Iron is essential for life and its amount in cells is controlled by the cell surface transferrin receptor (TfR)-mediated uptake of iron as transferrin-iron. TfR synthesis is regulated by interaction of the iron regulatory protein (IRP) with the iron-responsive element (IRE) present on the 3’-untranslated region of TfR mRNA. IRPs serve as sensors of cellular iron [78]. The cellular oxidative damage caused by reactive oxygen species and reactive nitrogen species is critically controlled by cellular iron homeostasis [79]. Exposure to H_2_O_2_, enhances the expression of TfR mRNA, where TfR appears to be the link between oxidative stress and TfR-mediated iron uptake. Transferrin (Tf) is the iron carrier glycoprotein for all normal cells of mammalians. In circulation, it is saturated to around 30% in adults. Thus, allowing for the chelation of iron radicals released after cell injury. In the CNS, Tf is synthesized by OLs and choroid plexus. Iron (Fe) is found in OLs and myelin in high density and both iron and Tf are required for myelin production [80]. As discussed by Takeda and collaborators and other authors, excess and/or free iron can be cytotoxic, by catalyzing the production of hydoxyl radicals from hydrogen peroxide [81]. Increased iron in the brain has been found in neurodegenerative diseases, e.g. Alzheimer’s disease and Parkinson’s disease [82,83] where oxidative stress has been proposed as a pathogenic mechanism of neurodegeneration. Conversely, brain Tf levels decrease with age and the decrease is dramatic when Alzheimer’s and Parkinson’s disease are superimposed on the aging process [84]. Therefore, stoichiometry of the iron related proteins Tf, ferritin and transferrin receptor (TFR) is essential to maintain CNS function. Upon OLPs and or myelin breakdown free iron is released. In our experimental model, Tf may act as a transient chelator while “Ferritin”, the iron storage protein, is synthetized helping prevent oxidative stress and lipid peroxidation.

It is important to assert that we have previously shown synergistic effects of TSC1on myelination in animal models of dys-myelination (inherited myelin deficiency) [36] and adult CNS progressive demyelination [37], as well as in a spinal cord severe crush mouse model. In the present work, we showed that a single dose of TSC1 was necessary and sufficient to protect OLPs and nestin-expressing progenitors that were present at the time of insult by NMDA. Thus, TSC1 in the case of glutamate excitotoxicity also acts as a neuroprotective agent. A search for therapies to enhance repair of the immature brain has extended for more than two decades and it continues. We believe that the present work is one step forward towards the common goal of repairing the white matter of the premature neonate that in turn will lead to the improvement of long-term motor and cognitive outcomes. To our knowledge TSC1 is the only treatment that aims at the mobilization of endogenous stem/progenitor cells to replace lost or unhealthy OL aiming at restoration of CNS function by myelination in the premature neonate.
